# Ludwig Boltzmann Cluster Oncology (LBC ONC): first 10 years and future perspectives

**DOI:** 10.1007/s00508-018-1355-7

**Published:** 2018-07-13

**Authors:** Peter Valent, Emir Hadzijusufovic, Thomas Grunt, Heidrun Karlic, Barbara Peter, Harald Herrmann, Gregor Eisenwort, Gregor Hoermann, Axel Schulenburg, Michael Willmann, Rainer Hubmann, Medhat Shehata, Edgar Selzer, Karoline V. Gleixner, Thomas Rülicke, Wolfgang R. Sperr, Brigitte Marian, Michael Pfeilstöcker, Hubert Pehamberger, Felix Keil, Ulrich Jäger, Christoph Zielinski

**Affiliations:** 1 Ludwig Boltzmann Cluster Oncology, Vienna, Austria; 2 Department of Internal Medicine I, Division of Hematology & Hemostaseology, Medical University of Vienna, Waehringer Guertel 18–20, Vienna, Austria; 3 Department/Clinic for Companion Animals and Horses, Clinic for Small Animals, Clinical Unit of Internal Medicine, University of Veterinary Medicine Vienna, Vienna, Austria; 4 Department of Internal Medicine I, Division of Clinical Oncology, Medical University of Vienna, Vienna, Austria; 5 Hanusch Hospital, Vienna, Austria; 6 Department of Radiation Oncology, Medical University of Vienna, Vienna, Austria; 7 Department of Laboratory Medicine, Medical University of Vienna, Vienna, Austria; 8 Department of Internal Medicine I, Stem Cell Transplantation Unit, Medical University of Vienna, Vienna, Austria; 9 Department of Companion Animals and Horses, Clinic for Internal Medicine and Infectious Diseases, University of Veterinary Medicine Vienna, Vienna, Austria; 10 Institute of Laboratory Animal Science, University of Veterinary Medicine Vienna, Vienna, Austria; 11 Department of Internal Medicine I, Institute of Cancer Research, Medical University of Vienna, Vienna, Austria; 12 Department of Dermatology, Medical University of Vienna, Vienna, Austria

**Keywords:** Cancer stem cells, Leukemic stem cells, Targeted therapy, Precision medicine, Immunotherapy

## Abstract

In 2008 the Ludwig Boltzmann Cluster Oncology (LBC ONC) was established on the basis of two previous Ludwig Boltzmann Institutes working in the field of hematology and cancer research. The general aim of the LBC ONC is to improve treatment of hematopoietic neoplasms by eradicating cancer-initiating and disease-propagating cells, also known as leukemic stem cells (LSC) in the context of leukemia. In a first phase, the LBC ONC characterized the phenotype and molecular aberration profiles of LSC in various malignancies. The LSC phenotypes were established in acute and chronic myeloid leukemia, in acute lymphoblastic leukemia and in chronic lymphocytic leukemia. In addition, the concept of preleukemic (premalignant) neoplastic stem cells (pre-L-NSC) was coined by the LBC ONC and was tested in myelodysplastic syndromes and myeloproliferative neoplasms. Phenotypic characterization of LSC provided a solid basis for their purification and for the characterization of specific target expression profiles. In a second phase, molecular markers and targets were validated. This second phase is ongoing and should result in the development of new diagnostics parameters and novel, more effective, LSC-eradicating, treatment strategies; however, many issues still remain to be solved, such as sub-clonal evolution, LSC niche interactions, immunologic control of LSC, and LSC resistance. In the forthcoming years, the LBC ONC will concentrate on developing LSC-eradicating strategies, with special focus on LSC resistance, precision medicine and translation of LSC-eradicating concepts into clinical application.

## Introduction and historical overview

During the past 20 years, cancer-initiating stem cells (CSC) have been extensively analyzed in basic science and in translational medicine [1–7]. In contrast to their more mature daughter cells, CSC have the capacity to propagate a neoplasm (cancer) *in vivo* for unlimited time periods. In line with this definition, CSC exhibit self-renewal and long-term disease-propagating capabilities. In addition, CSC have the ability to undergo asymmetrical cell division and subsequent differentiation to form the bulk of “more mature” cells in a malignancy. The concept of CSC was first established in myeloid leukemia where CSC are also termed leukemic stem cells (LSC) [8–10]. In certain disease models, such as chronic myeloid leukemia (CML), the clonal hierarchy and LSC dependence are obvious predominant features [11–13].

Over the years, the CSC hypothesis has also been tested in diverse forms of solid tumors and in melanoma patients [6, 7, 14–22]. To a degree, clonal evolution and stem cell hierarchies are also demonstrable in these malignancies [14–22]; however, in advanced (metastatic) cancer and melanoma as well as in most forms of acute leukemia, the stem cell hierarchy in (most of) the dominant clones is gradually (and often rapidly) diminishing [23–28]. Moreover, in advanced neoplasms, neoplastic stem cells are heterogeneous populations of cells, and depending on the stage and type of neoplasm, “stemness” may be or may become a reversible or newly acquired feature of (certain subsets of) neoplastic precursor cells [20–28].

Clinically relevant questions and issues in CSC research are the discovery of more or less specific (defining, diagnostic and/or prognostic) markers and oncogenic pathways in CSC/LSC, the identification and characterization of molecular targets in these cells, and the stem cell-eliminating capacity of various targeted drugs and drug combinations [4, 5, 7, 29–34]; however, whereas a number of different markers, targets, and target pathways have been identified in CSC and LSC, numerous questions remain, such as the origin and clonal evolution of these cells, mechanisms contributing to the multiple forms of stem cell resistance, and the interactions of neoplastic stem cells with the specific microenvironment (stem cell niche) and the immune system (Table 1; [35–40]).

**Table 1:** Major issues in cancer/leukemic stem cell research

Origin of neoplastic/leukemic stem cells (NSC/LSC)
Definition of NCS/LSC (biology and function)
Terminology/nomenclature: e.g., pre-L-NSC
Classification of NSC: premalignant versus malignant
Comparing various disease models: NSC, LSC, CSC.
Selecting the optimal stem cell assays and models
Optimal mouse xenograft model to study NSC engraftment
Selection of optimal purification protocols
Impact of the stem cell niche
Impact of the immune system
Markers and targets of NCS (LSC and CSC)
Effects of targeted drugs on NSC, LSC, and CSC
Definition of eradication and cure in NSC context
Eradication of CSC/LSC versus eradication of all NSC
Mechanisms of drug resistance of NSC, LSC and CSC
How to overcome drug resistance of NSC/LSC/CSC

*CSC* cancer stem cell(s); *LSC* leukemic stem cell(s); *pre-L-NSC* preleukemic neoplastic stem cell(s)

**Fig. 1:**
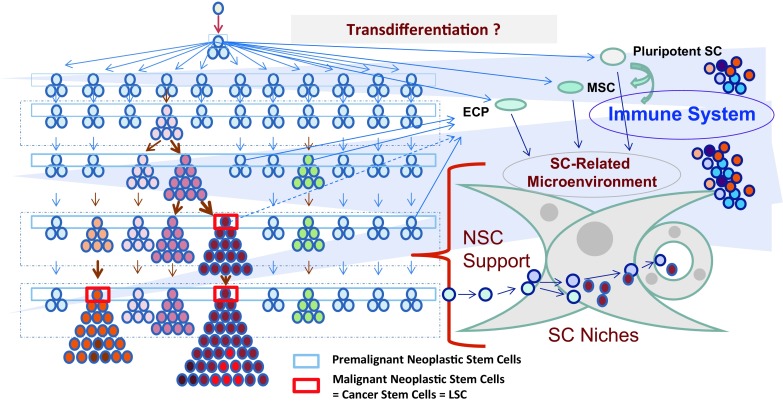
Evolution of neoplastic disorders and impact of the microenvironment. In an early phase of cancer evolution, normal stem cells acquire early lesions and thereby transform into self-renewing neoplastic stem cells (*blue rectangle boxes*). These premalignant neoplastic stem cells are slowly cycling or dormant cells that persist and slowly acquire additional pro-oncogenic lesions and hits (color change) in distinct sub-clones. The resulting diversification into many different sub-clones is a constantly ongoing process. In a next step, the stem cells of one or more of these premalignant sub-clones acquire critical combinations of oncogenic hits and thereby an enhanced proliferative potential and the capacity to produce an overt neoplasm. Finally, these stem cells and their sub-clones acquire additional pro-oncogenic lesions and convert into fully malignant neoplastic stem cells (*red rectangle boxes*) producing an overt cancer. In the context of a solid tumor, these cells are then called cancer stem cells (*CSC*), and in the context of leukemia, these cells are termed leukemic stem cells (*LSC*). Cancer/leukemia evolution is triggered by the genetic background, a number of different exogenous factors (viruses, toxins, etc.), epigenetic events, the microenvironment and the immune system. The stem cell-related microenvironment, also called stem cell (*SC*) niche, has been implicated in stem cell survival, stem cell homing and stem cell resistance. In certain neoplasms, some of the micro-environmental cells may be derived from clonal cells through transdifferentiation. The immune system has been implicated in immunosurveillance; however, in an overt malignancy, neoplastic stem cells have the capacity to escape immunosurveillance

An important aspect is that during malignant progression, proliferation of CSC/LSC gradually shifts from asymmetrical to (more and more) symmetrical cell divisions, resulting in a huge accumulation of cells possessing stem cell features. During this process of stem cell expansion, more and more somatic aberrations and epigenetic changes are acquired by the dominant sub-clone(s) (Fig. 1; [38–46]). In advanced neoplasms and acute leukemia, CSC/LSC indeed exhibit diverse combinations of molecular lesions and oncogenic pathways that lead to an increasing tumorigenic potential [43–50]. These processes are triggered by the genomic instability and plasticity. Sub-clone formation, expansion and diversification of clonogenic cells may also be regulated by the disease-specific microenvironment (niche) and the immune system (Fig. 1). There is also growing evidence that extensive sub-clone formation and diversification essentially contributes to acquired drug resistance of CSC/LSC, which occurs during disease evolution [32–34, 42–50].

Research on LSC developed rapidly in various countries in the Western world over the past two decades. In Austria, first attempts were made to establish robust xenotransplantation models for primary, patient-derived cells between 2000 and 2005. An essential step forward was the introduction of more permissive immunodeficient mice, including the NSG mouse model that has been used successfully in various leukemia models over the past 15 years.

The Ludwig Boltzmann Cluster Oncology (LBC ONC) was established in 2008 by merging two institutes working in the field of hematology and cancer research, the Ludwig Boltzmann Institute for Leukemia Research and Hematology and the Ludwig Boltzmann Institute for Clinical and Experimental Oncology. The newly formed LBC ONC was soon able to attract a group of capable scientists and to establish a number of fruitful collaborations with local and international experts. During the past 10 years, the LBC ONC expanded substantially and launched a series of projects on LSC in various disease models. In these projects, members of the LBC ONC were able to delineate the phenotype and biology of LSC and to develop theoretical concepts around these cells. A major concept propagated by the LBC ONC is that neoplastic stem cells (NSC) can be divided into premalignant (preleukemic) neoplastic stem cells (pre-L-NSC) and fully malignant (leukemic/cancerous) neoplastic stem cells [32, 43–46]. In the context of leukemia, these malignant NSC are called LSC.

Members of the LBC ONC also organized a series of international meetings and conferences in the past 10 years. In the current article, the aims, achievements and future perspectives of the LBC ONC are summarized.

## Major aims of the LBC ONC

The general aim of the LBC ONC is to identify and characterize LSC in myeloid and lymphoid neoplasms, to identify clinically relevant (diagnostic and/or prognostic) markers and therapeutic targets (target expression profiles) in these cells, and to examine the effects of various targeted drugs and drug combinations on growth and survival of LSC. In the past 10 years, the LBC ONC has made considerable progress in the phenotyping and characterization of LSC and pre-L-NSC in various leukemia models and preleukemic conditions and has started to validate a number of promising markers and therapeutic targets in these cells. The ultimate aim of the LBC ONC is to establish a solid basis for the development of novel LSC-eradicating treatment concepts. The related long-term vision of the research program of the LBC ONC is to establish improved, potentially curative approaches for human leukemias by applying drugs and drug combinations or immunotherapies that can eliminate LSC and pre-L-NSC in these malignancies, either directly or by targeting the LSC-supporting niche and/or by modulating immunologic mechanisms, such as checkpoint expression, and other processes contributing to LSC resistance.

A general strategic aim of the LBC ONC is to provide a multidisciplinary platform for interactive, cooperative research on NSC/LSC in Vienna and Austria. A related specific aim is to assist in the initiation of specific collaborations in the field of LSC research and to intensify and assist ongoing collaborations with national and international partners and networks working in the field of LSC. To reach these goals, the LBC ONC has established a series of collaboration meetings and platforms, with the aim to discuss novel developments in the field and new results in ongoing projects and collaborations. Moreover, the LBC ONC organized a series of international meetings and conferences on CSC/LSC in the past 10 years. Another important aim of the LBC ONC is to educate young scientists interested in LSC research, to promote junior group leaders in the field, and to motivate clinical colleagues to translate LSC concepts and results into clinical application. With respect to clinical trials and translational projects, the LBC ONC exploits a strong local network of clinical groups working in the field of leukemia research and several different interactions between the cluster core group, the Medical University of Vienna and external partners, such as the Hanusch Hospital, where clinical hematology is a well-established focus, several national and international study groups, and various industrial partners. Moreover, the LBC ONC cooperates with the Comprehensive Cancer Center (CCC) of the Medical University of Vienna.

## Major topics and project lines

At the time of initiation in 2008, four project lines were launched: one focusing on myeloid neoplasms, one on lymphoid neoplasms, one on solid tumors, and one on skin cancer models and mastocytosis. Over the years, the cluster focused primarily on myeloid, lymphoid and mast cell neoplasms, following the recommendations of their scientific advisors and members of the evaluation board. Nevertheless, the LBC ONC also published results on putative CSC in solid tumors (colon adenoma and colon cancer) and putative melanoma stem cells [7, 22, 38, 51–54].

Each project line is based on 2 or more research groups and a series of interconnected projects on LSC. Based on evaluation reports and recommendations of the scientific advisory board (SAB) and depending on the developments in the field and available infrastructure, the LBC ONC decided to focus on certain disease models in each group of neoplasms. Among myeloid neoplasms, the LBC ONC is focusing on acute myeloid leukemia (AML), myelodysplastic syndromes (MDS), and chronic myeloid leukemia (CML). More recently, the LBC ONC has also started to work in the field of Ph-negative myeloproliferative neoplasms (MPN) and on chronic myelomonocytic leukemia (CMML). Within lymphoid neoplasms, the LBC ONC started to focus on chronic lymphocytic leukemia (CLL) and acute lymphoblastic leukemia (ALL). Later, the LBC ONC also examined putative stem cells and their response to targeted drugs in multiple myeloma [55]. Within the group of solid tumors, the LBC ONC started to focus on colon adenomas and colon carcinomas [51]; however, over time most of these projects had to be discontinued in the LBC ONC. Similarly, although the cluster published on melanoma-initiating cells [54], melanoma projects were discontinued over time. Within mast cell neoplasms, the LBC ONC started to characterize stem cells in aggressive systemic mastocytosis (ASM) and mast cell leukemia (MCL). More recently, the LBC ONC also started to characterize pre-L-NSC in indolent SM (ISM) and smoldering SM (SSM).

Overall, the LBC ONC characterized disease-initiating and propagating cells in diverse forms of acute (aggressive) malignancies in a first research phase. More recently, the LBC ONC also started to characterize preleukemic neoplastic stem cells (pre-L-NSC) in various indolent neoplasms, including MDS, MPN, CMML and ISM/SSM. In addition, the LBC ONC has started to characterize pre-L-NSC in prediagnostic stages of a malignancy and residual pre-L-NSC and LSC in patients who are in complete remission after drug therapy (e.g., chemotherapy or targeted drugs).

In each project line, the LBC ONC was able to organize constant access to primary patient-derived samples and established a robust biobank which was only possible to accomplish through the constant support of the Medical University of Vienna. In addition, the LBC ONC was able to establish robust xenotransplantation models through a strong essential collaboration with the University of Veterinary Medicine in Vienna. Based on this fruitful collaboration, the LBC ONC invited the University of Veterinary Medicine Vienna to join the cluster, and in 2017 this important institution indeed joined and became an official partner of the LBC ONC.

## Management, internal collaborations and core facility platforms

The LBC ONC was established in 2008 by merging two previous Ludwig Boltzmann Institutes, one located at Hanusch Hospital and the other at the Medical University of Vienna. In 2017, the University of Veterinary Medicine Vienna joined as a third partner institution. The three academic partners are interconnected via a number of well-established and fruitful scientific collaborations in the field of leukemia research, translational hematology and clinical hematology. In addition, based on fruitful collaborations on mast cell neoplasms between the University of Veterinary Medicine Vienna and the Medical University of Vienna, the new emerging field of comparative oncology has become a new focus in the LBC ONC.

The LBC ONC is based on a robust communication system that promotes continuous networking and scientific collaborations and discussion. Internal communication in the LBC ONC and interactions with external partners are coordinated and guided by the administration team of the cluster. This team is instrumental for the management of the LBC ONC and is coordinated by the administrative coordinator, Emir Hadzijusufovic and his team. Members of the LBC ONC meet regularly in weekly progress report meetings at the Medical University of Vienna. These meetings are incorporated in the Vienna Cancer Stem Cell Club (VCSCC) [56] and represent a suitable (permanent) platform for fruitful scientific discussion and collaborations. New projects are introduced and ongoing projects are discussed on a regular basis (at least monthly) in these meetings. In collaboration with the VCSCC, the LBC ONC also hosts a weekly journal club and a seminar series where international experts are invited to Vienna. Finally, the LBC ONC organized a number of smaller and larger international scientific meetings on CSC/LSC, including several major working conferences (Table 2).

**Table 2:** International conferences and meetings organized by the LBC ONC (2008–2018)

Title of conference/workshop^a^	Place/year	Publications^b^
Second Vienna Cancer Stem Cell Meeting	Vienna 2008	–
Year 2011 Working Conference on Cancer Stem Cells	Vienna 2011	[43, 46]
10 Year Jubilee Meeting of the VCSCC, Billroth Haus, Vienna	Vienna 2012	[56]
Ph^+^ CML: from LSC eradication to cure	Vienna 2014	–
From cellular basis and targets to targeted therapies	Vienna 2015	[95]
Classification and nomenclature of clonal conditions	Vienna 2015	[96]
Workshop on mast cells and mastocytosis	Vienna 2015	[87]
Biology, classification and therapy of basophil disorders	Vienna 2016	[97]
Workshop on MDS and pre-MDS conditions	Vienna 2016	[98]
Workshop comparative oncology—Mast cell neoplasms	Vienna 2017	Submitted
Workshop on erythropoiesis and erythroid disorders	Vienna 2017	Submitted
10 Year Jubilee Meeting of the LBC ONC	Vienna 2018	This manuscript
Working Conference on Chronic Myelomonocytic Leukemia	Vienna 2018	Planned

^a^In each conference, the first day included an education session open to the public and free of registration (no registration fee)

^b^Position papers were prepared to summarize the event and the most important conference outcomes in form of an overview and/or in form of consensus statements. VCSCC, Vienna Cancer Stem Cell Club

In 2017 an updated contract that regulates the interactions between partners and their financial supporters in the LBC ONC was signed. Based on this new contract, a new board of partners assembling major representatives of all partner institutions was established. The board of the LBC ONC meets two times per year to discuss the general strategies and conceptual issues in the LBC ONC and to report about achievements and plans as well as the budget of the cluster. The LBC ONC has also established a scientific advisory board (SAB) consisting of 3 international experts in the field. The SAB members visit the LBC ONC once per year and provide critical input and important scientific advice to cluster coordinators as well as to postdocs and students working on LBC ONC projects.

During the first 10 years, the LBC ONC has established seven core facility platforms (CF-PF) in collaboration with their academic partners. These CF-PF include a management and administration CF-PF, a flow cytometry and LSC-sorting CF-PF, an omics CF-PF, a mouse (NSG) xenotransplantation CF-PF, an education CF-PF, a clinical study CF-PF and a CF-PF for viral-mediated gene delivery and the establishment of related cell lines. The CF-PF are interconnected with each other and with all ongoing cluster projects and provide an essential technology network and basis for the successful research in the three project lines of the LBC ONC.

## Established collaborations and networking

The LBC ONC established numerous collaborations with local, national and international groups and institutes working in the field of hematology and cancer research and with various academic organizations and societies. Important local collaborating institutions include the Children’s Cancer Research Institute (CCRI), the Institute for Molecular Pathology (IMP), the Center for Molecular Medicine (CeMM) of the Austrian Academy of Science, the Ludwig Boltzmann Institute for Cancer Research (LBI-CR), the Ludwig Boltzmann Cluster for Cardiovascular Research, and the Ludwig Boltzmann Institute for Osteology. Several of these collaborations are embedded in strong scientific networks that were formed on the basis of the VCSCC and the LBC ONC. One good example is a special research program (SFB) on MPN that has been initiated by the Medical University of Vienna and is coordinated by members of the LBC ONC. Another good example is the international networking on MDS where members of the LBC ONC from the Hanusch Hospital and the Medical University of Vienna worked together with their international partners and essentially contributed to numerous studies [57–60]. The LBC ONC has also established important collaborations with certain industrial partners interested in translational and clinical hematology and LSC research. Based on mutual interest, several of these collaborations have been intensified in the recent past. Whereas the LBC ONC is able to test new innovative drugs against LSC, the industrial partners can learn whether their drugs may indeed have a curative (LSC-eliminating) potential.

## Major events and conferences

In 2008, members of the LBC ONC and the VCSCC co-organized the second Cancer Stem Cell Meeting in Vienna (Table 2). Several pioneers in the field of CSC/LSC research participated and the LBC ONC was able to present key researchers, postdocs and students as well as the strategic research plan to interested visitors and to the international community. In 2011, the LBC ONC and the VCSCC organized the Year 2011 Working Conference on CSC (Table 2). In this meeting, a panel of authorities in the field discussed various aspects of CSC/LSC research, including terminologies, the biology and heterogeneity of NSC (pre-C-NSC/pre-L-NSC vs. CSC/LSC) as well as limitations of these concepts, and the assays that have to be applied to detect and quantify CSC/LSC [43]. As a result a new classification of NSC was proposed by the faculty. Based on this proposal, neoplastic stem cells (the umbrella term) are divided into premalignant (in leukemia context preleukemic) neoplastic stem cells (pre-L-NSC or pre-C-NSC) and malignant (leukemic) neoplastic stem cells (Table 3; [43]). Premalignant NSC can produce a premalignant/preleukemic neoplastic condition (e.g., MDS or MPN) but not a fully transformed malignancy (e.g. AML). Over time, pre-L-NCS can transform into fully malignant LSC, depending on additional lesions and hits that are acquired by these cells [43, 46]. Stem cell evolution is a multistep process that takes years or even decades, and is propagated (by driver and passenger mutations) in one, more, or many different (separately developing) sub-clones (Fig. 1). This new concept and the related classification of neoplastic stem cells may explain a number of different clinical phenomena, such as the extensive plasticity and heterogeneity of primary and secondary cancer lesions, lineage switches of cancer cells, or the different biology and resistance of relapsing disease. The faculty delivered consensus statements and the proposed classification in 2012 [43]. In October 2012, the VCSCC celebrated its 10 Year Jubilee in the form of a Scientific Workshop on CSC/LSC in the “Billroth Haus” in Vienna (Table 2). Members of the LBC ONC co-organized this event and in April 2018, members of the LBC ONC celebrated the 10 Year Jubilee of the cluster in a research meeting in Vienna.

**Table 3:** Classification of stem cells in preleukemia and leukemia patients and clinical implications

Stem cell class	Major clinical implications
Normal stem cells	Can be detected in patients with leukemia and preleukemic conditions^a^ Basis of curative drug therapies (regeneration of normal BM)
Pre-L-NSC	(1) Can produce a preleukemic neoplastic condition, such as MDS or MPN
	(2) Can transform into LSC and thereby trigger a full-blown leukemia, such as AML
	(3) Often escapes interventional therapy, including poly-chemotherapy and targeted therapies
	(4) Can cause a late relapse after therapy^b^
	(5) Can increase the risk of thromboembolic events (when ARCH mutations are expressed)
LSC	Produce an overt leukemia; are often resistant against conventional chemotherapy and can cause early relapse or drug-resistant disease
	These cells need to be eradicated to achieve a complete remission (CR) after chemotherapy. In many patients long-term CR is achieved^b^

*BM* bone marrow *Pre-L-NCS* pre-leukemic neoplastic stem cells, *MDS* myelodysplastic syndrome, *MPN* myeloproliferative neoplasms, *AML* acute myeloid leukemia, *ARCH* age-related clonal hematopoiesis

^a^Preleukemic conditions are all clonal processes that may precede a leukemia; preleukemic neoplasms are all overt hematopoietic neoplasms that can precede a leukemia

^b^When all Pre-L-NSC and LSC can be eradicated therapy is curative and no relapse can occur. When all LSC but only some or most Pre-L-NSC can be eradicated, the patient may or may not enter long-term CR, depending on the progression potential of the residual Pre-L-NSC

## Major concepts around LSC and pre-L-NSC

Cancer evolution is an extremely complex and long-lasting process that can essentially be divided into prediagnostic, premalignant and malignant phases (stages) of the disease. The same holds true for leukemic disorders, where preleukemic neoplasms and overt leukemia have to be distinguished. As mentioned before, NSC can also be divided into premalignant neoplastic stem cells (pre-C-NCS or pre-L-NSC) and fully malignant stem cells (CSC/LSC) [43, 46]. Whereas pre-L-NSC can produce a preleukemic neoplasm, such as MDS, only LSC can produce an overt leukemia (Table 3). The concept of pre-L-NSC and LSC has been confirmed in various preclinical models and clinical studies and has major implications for the development of novel diagnostic and prognostic concepts and novel therapies.

A major discovery has been that pre-L-NSC are frequently detectable in older, apparently healthy individuals. In fact, age-related somatic mutations (ARCH), also known as clonal hematopoiesis of indeterminate potential (CHIP) [61–64], are considered to be produced by pre-L-NCS, provided that the somatic clone persists. Moreover, it has been shown that these early clones can acquire additional mutations over time and that these additional mutations can contribute to progression to an overt neoplasm or a relapse [65, 66]. It has also been described that after successful chemotherapy or successful targeted therapy, when the dominant clones are no longer detectable, early pre-L-NSC may persist and may be detectable by testing blood cells for the presence of CHIP/ARCH mutations (Table 3; [67–69]). Finally, CHIP/ARCH mutations have been associated with a high risk to develop atherosclerosis and related (life-threatening) thromboembolic events [63, 69, 70]. This unexpected observation may explain why many (if not most) blood cell disorders are associated with an increased risk of thromboembolic events.

## Phenotypic characterization of LSC and LSC resistance

During the past 10 years, the LBC ONC has been able to characterize the phenotype of putative NSC/LSC in various hematopoietic neoplasms. The LSC were identified in patients with Ph^+^ CML, AML, CLL, Ph^+^ ALL, Ph^−^ ALL and MCL [71–80]. Putative NSC (pre-L-NSC) were identified in patients with MDS, systemic mastocytosis and Ph^−^ MPN [77, 79]. As assessed by gene array and antibody-based screening, a number of cell surface antigens, such as roundabout-4 (ROBO4), the IL-2 receptor alpha chain (CD25), dipeptidyl-peptidase IV (DPPIV = CD26), Siglec-3 (CD33) or Campath-1 (CD52), were identified on neoplastic stem cells. An interesting observation was that in all overt leukemias, including even CLL, the NSG mouse-engrafting NSC/LSC apparently resided in a CD34+ sub-fraction of the neoplastic clone (Table 4). Another interesting observation was that several markers identified on LSC are also detectable on stem cell populations in preleukemic conditions (pre-L-NSC) and even also in non-hematologic (solid) tumors. For example, CD26 and CD133 are expressed on putative colon CSC as well as on CML LSC [53, 72]. Finally, several of the newly identified surface markers on NSC/LSC may serve as diagnostic indicators or as molecular targets. A summary of surface markers and targets detected on pre-L-NSC and LSC in hematologic neoplasms is shown in Table 4. In Ph^+^ CML, the NSG mouse-engrafting LSC reside within a sub-fraction of CD34^+^/CD38^−^ cells that aberrantly co-express CD25, CD26 and IL-1RAP (Table 4; [72]). In addition, these cells co-express increased amounts of CD33, CD44, CD56 and CD90 when compared to normal bone marrow stem cells. In AML, the putative CD34^+^/CD38^−^ LSC display, among other antigens, CD25, CD33, CD44, IL-1RAP and CD371 (CLL-1; Table 4). In Ph^+^ ALL, CD34^+^/CD38^−^ LSC often express CD25, CD26 and IL-1RAP, whereas in Ph^−^ ALL, LSC usually lack CD25, CD26 and IL-1RAP [80]. In patients with MDS, the putative CD34^+^/CD38^−^ pre-L-NSC often express CD25, CD33, and CD44 as well as CD52 [77, 81].

**Table 4:** Phenotype of leukemic stem cells (LSC) and identified targets^a^

Disease/neoplasm	Primary phenotype	Molecular targets^a^
AML (CD34^+^)^b^	CD34^+^/CD38^−^; CD34^+^/CD38^+^	CD33, CD44, CD52, KIT,
	CD34^+^/CD38^−^/CD123^+^	FLT3, HO-1, BRD4, MYC
	CD34^+^/CD38^−^/CD25^+^	
	CD34^+^/CD38^−^/CLL-1^+^	
Ph^+^ CML	CD34^+^/CD38^−^/CD25^+^/CD26^+^/	CD33, CD44, CD52, KIT,
	CD56^+^/CD93^+^/L-1RAP^+^	HO-1, MCL-1, AURKA/B
Ph^+^ ALL	CD34^+^/CD38^−^; CD34^+^/CD38^+^	CD19, CD22, CD33, CD52
	CD34^+^/CD38^−^/CD25^+^/CD26^+^	HO-1, BLC-2, MCL-2
Ph^−^ ALL	CD34^+^/CD38^−^; CD34^+^/CD38^+^	CD19, CD22, CD33, CD52
	CD34^+^/CD38^−^/CD25^+^	HO-1, BCL-2, MLC-1
CLL	CD34^+^/CD19^+^/CD5^+^	CD20, CD23, CD44, CD52
		Notch1/2, PTEN, PI3K
ASM/MCL	CD34^+^/CD38^−^/CD123^+^	CD33, CD44, CD52, KIT
		HO-1, FES, AURKA/B,

Cell surface expression of markers and targets on LSC was determined by multi-color flow cytometry

*AML* acute myeloid leukemia, *HO-1* heme oxygenase-1, *CML* chronic myeloid leukemia, *ALL* acute lymphoblastic leukemia, *CLL* chronic lymphocytic leukemia, *PI3K* phosphoinositide-3-kinase, *ASM* aggressive systemic mastocytosis, *MCL* mast cell leukemia, *AURK* aurora kinase

^a^The Table includes a few selected molecular targets identified in LSC by members of the LBC ONC in the past 10 years

^b^In AML, both the CD34^+^/CD38^−^ and CD34^+^/CD38^+^ subsets of CD34^+^ cells contain LSC

During validation, members of the LBC ONC were interested to learn (i) about the mechanism(s) underlying abnormal or even selective aberrant expression (compared to normal stem cells) of markers and targets on LSC, (ii) about the functional pathogenetic role of these antigens, and (iii) about possibilities to employ these antigens as suitable diagnostic or prognostic markers or as molecular targets of therapy. For example, CD25 was found to be a STAT5-dependent negative regulator of growth of CML LSC [74]. The antigen CD26, also known as DPPIV, was identified as an enzyme that is involved in the mobilization of CML LSC out of the stem cell niche which may explain the huge spread of LSC into extramedullary organs in CML [72]. Another example is CD52. Expression of this antigen is triggered by the RAS pathway and often upregulated on LSC in MDS and AML patients exhibiting the 5q-anomaly [77].

During the past 5 years, members of the LBC ONC have explored the mechanisms underlying the interactions between LSC and the organ and disease-specific microenvironment, the so-called stem cell niche. As mentioned before, CD26 is an important regulator of LSC-niche interaction. Another observation has been that certain LSC-niche interactions contribute to drug resistance. Therefore, the LBC ONC has also explored the mechanisms of various targeted drugs on niche cells. For example, certain tyrosine kinase inhibitors directed against BCR-ABL1 and other (vascular) targets, like nilotinib, suppress endothelial cell proliferation and angiogenesis and may thus impact on the vascular stem cell niche [69].

Finally, certain cell surface antigens on pre-L-NSC and/or LSC may be associated with or even trigger, drug resistance. Among these are the multidrug-resistance gene product MDR-1 (CD243), “the don’t eat me” checkpoint receptor CD47, and the T cell-PD1-interacting checkpoint molecule PD-L1 (CD274). These antigens are frequently detectable on LSC in AML, CML and other hematopoietic neoplasms, and are regulated by specific pathways. For example, expression of PD-L1 on LSC depends on a BET-MYC pathway that can be induced by the oncogenic machinery and/or the cytokine storm involving interferon-gamma [82]. Currently, members of the LBC ONC are exploring the mechanisms underlying the well-known resistance of LSC against various targeted and conventional drugs and how LSC-niche and LSC-immune cell interactions contribute to the intrinsic and acquired forms of resistance of LSC.

## Clinical translation of LSC concepts: examples and status 2018

During the past few years, members of the LBC ONC were able to translate several of the concepts that were developed in their cluster projects into clinical application. These include novel diagnostic approaches, prognostication, and therapies. In the following paragraphs, a few highlighting examples are provided.

*1. Diagnostic LSC phenotyping*: during the past 5 years members of the LBC ONC have established LSC expression profiles in myeloid and lymphoid leukemias by testing antibody combinations in larger cohorts of patients. During validation, aberrantly expressed markers were also examined for their specificity and sensitivity. As a result, several markers have been introduced into application in clinical practice. In CML, the recommended LSC markers are CD25, CD26 and IL-1RAP [72–74]. In most patients with chronic phase CML, all three antigens are detectable on CD34^+^/CD38^−^ stem cells; however, in advanced CML one or more of these antigens may not be detectable on LSC. Even in chronic phase CML, LSC may sometimes lack one of these antigens. Therefore, all three markers should be applied in diagnostic phenotyping. The CCL-1 (CD371), a marker of AML LSC, is usually not expressed on CML LSC. In AML, CD34^+^/CD38^−^ LSC often display CD25 (in about 40% of patients) and/or CCL-1 (CD371; in about 30% of patients). In addition, IL-1RAP may be detected on AML LSC. By contrast, AML LSC usually stain negative for CD26. Normal bone marrow stem cells usually lack all these LSC-related surface antigens; however, some of these antigens, such as CD25, may be expressed on early preleukemic neoplastic CD34^+^/CD38^−^ stem cells (pre-L-NSC). For example, in patients with MDS, the putative CD34^+^/CD38^−^ pre-L-NSC often display CD25. Despite these encouraging results, diagnostic LSC phenotyping needs further validation in forthcoming studies.

*2. Linking pre-L-NSC to age-related clonal hematopoiesis (ARCH)*: recent data suggest that ARCH is a persistent (and thus stem cell-derived) molecular signature of somatic mutations predisposing (a) for the development of clonal hematopoietic neoplasms and (b) for the occurrence of life-threatening cardiovascular events [63, 69, 70]. The name ARCH implies that the prevalence of these mutations increases with age. The alternative name for ARCH is clonal hematopoiesis with indeterminate potential (CHIP) which implies that the final outcome is uncertain; however, it is well known that several of these patients develop a myeloid neoplasm, and it is also established that in patients with myeloid neoplasms the rate of cardiovascular events is relatively high. Therefore, analysis of patients with myeloid neoplasms for the presence of ARCH/CHIP mutations will soon be regarded as the standard. Even after successful debulking of all dominant sub-clones, ARCH/CHIP mutations may still be detected [66–69]. Based on concepts developed in the LBC ONC, these residual clones may be derived from pre-L-NSC and recent data suggest that carriers of ARCH/CHIP after therapy may indeed bear an increased risk to develop vascular events [69].

*3. Clinical translation of KIT-targeting drugs in advanced mast cell neoplasms*: KIT acts as a major and largely selective driver in systemic mastocytosis (SM). This neoplasm is defined by uncontrolled expansion and accumulation of neoplastic mast cells, certain clinical and molecular characteristics, and the transforming *KIT* mutation D816V. Whereas in indolent SM (ISM) the prognosis is excellent (normal life expectancy), patients with advanced SM, including aggressive SM (ASM) or MCL have a poor prognosis. Based on the specificity of KIT for SM, the LBC ONC focused research on this molecular target. In earlier studies, members of the LBC ONC were able to show that the *KIT* mutation D816V confers resistance against imatinib, and that this resistance can be overcome by a few novel TKIs. Among these, midostaurin was found to exert profound effects on the autonomous growth of KIT-mutated neoplastic mast cells [83]. In addition, members of the LBC ONC were able to show that midostaurin blocks mediator secretion in mast cells [84, 85]. Based on these observations and first pilot case reports, a global trial examining the effects of midostaurin in patients with advanced SM was conducted. In this study, the overall response rate was 60% despite the poor risk of the study group. Median overall survival was 28.7 months and the median progression-free survival amounted to 14.1 months [86, 87]. Among the 16 patients with MCL, the median survival time was 9.4 months and in some of the patients, long-lasting major responses were obtained [86, 87]. These results are superior compared to previous treatment options in advanced SM and MCL and have recently resulted in the approval of midostaurin for use in advanced SM by major health authorities [87]. Moreover, based on this study, several companies have recently started to develop novel more potent and more specific interventional drugs targeting KIT and various KIT mutant forms.

*4. Revival of antibody-based therapy in AML*: whereas in lymphoproliferative neoplasms the addition of targeted antibodies to induction poly-chemotherapy has clearly improved the overall treatment outcome and survival in these patients, no comparable antibody therapy is available in myeloid neoplasms. This is mainly due to the fact that the antibodies used thus far in myeloid leukemias, especially the CD33-directed antibody-conjugate gemtuzumab-ozogamicin (GO, mylotarg®) produced substantial myelotoxicity in clinical trials, which in turn can be explained by the stem cell-targeting effect of this drug. Notably, as mentioned before, CD33 is also expressed on normal stem cells. In addition, these antibodies, such as alemtuzumab (anti-CD52 antibody) can induce immunosuppressive effects by eliminating lymphocytes and monocytes. As a result, some of these agents, although very effective as anti-leukemic agents, such as GO, have been labeled as being “too toxic” and GO has even been (transiently) removed from the market as an AML therapy. During the past 10 years, members of the LBC ONC have shown that CD33 is indeed a robust target and that the levels of CD33 detectable on LSC in AML and CML are much higher (10–1000-fold) compared to expression levels on normal stem cells [71, 88, 89]. In addition, the *in vitro* effects of GO on LSC are much more pronounced than the effects on normal stem cells, providing a clear therapeutic window. More recently, several studies have shown that the myelotoxic effects of GO can be minimized by reducing the dose of GO and/or the numbers of applications. In addition, most of the larger studies and metanalyses have shown higher remission rates and a survival benefit for AML patients treated with GO [90, 91]. Based on these observations, mylotarg® (GO) is again available for the treatment of AML [91].

*5. Treatment of older patients AML patients with intensive (high dose) chemotherapy*: a special challenge in clinical hematology is the optimal choice of therapy for older patients (aged over 60 years) with AML. Many of these patients cannot tolerate intensive chemotherapy and most of them cannot be referred to hematopoietic stem cell transplantation (HSCT). On the other hand, less intensive therapy or therapy with hypomethylating agents often fails or is only effective for a certain time period. Even when these patients tolerate induction chemotherapy and reach complete remission, the (predicted) outcome is often poor, as many of these patients have secondary forms of AML or AML variants characterized by an unfavorable molecular or cytogenetic signature. Therefore, it is of utmost importance to develop new, improved treatment strategies for these patients. A straightforward approach would be to intensify the overall chemotherapy in these patients; however, due to the risk of severe infections and other side effects this strategy is questionable. Therefore, members of the LBC ONC and the Medical University of Vienna conducted a clinical trial exploring the clinical tolerability and efficacy of a special form of intensified consolidation after remission induction in older patients with AML [92]. The consolidation regimen was developed on the basis of a previous study showing that intermediate ARA‑C (cytosine arabinoside) is not only a highly effective but also a less toxic and extremely well-tolerated type of consolidation chemotherapy [93, 94]. In the trial conducted recently, AML patients ≥ 60 years in complete remission after induction therapy received fludarabine and ARA-C in consolidation cycle 1 and intermediate-dose ARA-C (IDAC) in cycles 2 through 4. In addition, pegfilgrastim (G-CSF) was applied: to examine the effect of pegfilgrastim on neutropenia and the time of hospitalization, the study compared cycles 2 and 4 where pegfilgrastim was given routinely from day 6 (IDAC-P) with cycle 3 where pegfilgrastim was only administered in case of severe infections and/or prolonged neutropenia. This regimen was not only found to be feasible but also to be less toxic and highly effective with superior response and survival data compared to (all known) previous trials conducted in older AML patients so far. The median duration of severe neutropenia was 7 days in cycle 2 (IDAC-P), 11.5 days in cycle 3 (IDAC), and 7.5 days in cycle 4 (IDAC-P; *p* < 0.05) [92]. The probability of survival after 5 years was 32%. Together, intensified consolidation can be administered in AML patients ≥ 60 years, and those who are <75 years were found to benefit from this therapy. Routine administration of pegfilgrastim seems important to shorten the time of neutropenia and hospitalization in AML. Whether this therapy approach can be regarded as a new standard of therapy in AML patients over 60 years, remains to be investigated in forthcoming, randomized clinical studies.

## Summary and future perspectives

The LBC ONC was established in 2008 with the aim to define phenotypic properties and target expression profiles in disease-initiating and propagating cells in various hematopoietic neoplasms and to improve anti-cancer therapy by eliminating these cells with more or less specific drugs and suitable (most effective) drug combinations. In addition, the LBC ONC has developed novel diagnostic tools and identified new prognostic stem cell markers. Several of these concepts have been translated from a preclinical stage into clinical application. The LBC ONC has also identified premalignant phases and cells in various myeloid malignancies. Finally, the LBC ONC has deciphered mechanisms underlying stem cell resistance and developed strategies to overcome resistance. In the project phase, the LBC ONC will continue to define the function and druggable vulnerabilities of neoplastic stem cells and will try to translate diagnostic, prognostic and stem cell-eradicating concepts into clinical application. In the context of new emerging therapeutic concepts, tools, and immunotherapies, there is hope that these translational approaches can lead to improved (curative) anti-neoplastic therapies in myeloid malignancies in the foreseeable future.
